# Raman spectroscopy of lymphocytes from patients with the Epstein–Barr virus infection

**DOI:** 10.1038/s41598-024-56864-y

**Published:** 2024-03-17

**Authors:** Magdalena Pietruszewska, Grażyna Biesiada, Jacek Czepiel, Malwina Birczyńska-Zych, Paulina Moskal, Aleksander Garlicki, Aleksandra Wesełucha-Birczyńska

**Affiliations:** 1 Faculty of Chemistry, Jagiellonian University, Gronostajowa 2, 30-387 Kraków, Poland; 2 Department of Infectious Diseases, Jagiellonian University, Medical College, Jakubowskiego 2, 30-688 Kraków, Poland; 3 The University Hospital in Kraków, Jakubowskiego 2, 30-688 Kraków, Poland

**Keywords:** Lymphocyte, B-cell, Epstein–Barr virus (EBV), Infectious mononucleosis, Raman microspectroscopy, Physical chemistry, Molecular medicine

## Abstract

In this study, Raman spectroscopy is applied to trace lymphocytes activation following contact with the Epstein–Barr virus (EBV) of the herpesvirus family. The biomarker of cell activation is found to be the 520 cm^−1^ band, indicating formation of immunoglobulins. The blood samples are obtained from patients diagnosed with infectious mononucleosis and treated at the University Hospital in Kraków. The lymphocytes’ Raman spectra are collected using a mapping technique, exciting samples with a 514.5 nm line of Ar^ +^ laser. Measurements are performed on the 1st, 4th, 6th, 12th and 30th day of hospitalization, until the patient has recovered. The highest intensity of the immunoglobulin marker is observed on the 4th day of hospitalization, while the results of the blood count of patients show the greatest increase in the number of lymphocytes at the beginning of hospitalization. No activated lymphocytes were observed in the blood of healthy volunteers. Some information is provided by the evaluation of B-cell activation by estimating the activated areas in the cells, which are determined by the presence of the Ig marker. The 900 cm^−1^ band and band around 1450 cm^−1^ are also analyzed as markers of the presence of the latent membrane protein, LMP2A (and 2B), of the EBV viral protein. The anomalous degree of depolarization observed in B-cells in the course of EBV infection appears to be due to the influence of a virus protein, disrupting BCR signal transduction.

## Introduction

Vibrational spectroscopy is currently used to study the molecular structures of biological systems by examining the individual components of these usually complex systems and identifying functional groups, which are markers of the state of studied systems. In this way, the determination of the characteristics and changes in the selected cells of patients diagnosed with the disease can be carried out through a spectroscopic view, which is selective to some extent, due to the physico-chemical basis of the phenomenon of light scattering^1,2^. Raman spectroscopy thus provides an insight into the molecular ordering of cells and tissues, facilitating analysis and early recognition of changes, which may provide an insight into potential medical applications^3,4^. In this sense, the cells of a living organism, or rather some moieties of cells, the vibrations of which are sensitive to chemical changes, become sensory elements^1^. This is due to the polarizability properties of individual functional groups that build cells. The chemical structure of biomolecules determines their polarizability properties, and thus, affects the intensity of Raman bands and even the observability of certain bands that can be selected as markers of ongoing processes. Monitoring changes provides an opportunity to consider phenomena from a different perspective other than analytical methods in biochemistry, biology or medicine. The key advantage of Raman spectroscopy is the ease of sample preparation, without the need for additional reagents or staining and its ability to detect changes at the molecular level at a very early stage of the disease, which other laboratory tests may not be sensitive enough to detect^5^.

Lymphocytes are among the cells that were studied successfully by Raman spectroscopy^6–9^. All these advantages render new technologies using Raman methods interesting in the study of viruses and virus-infected cells^10^. It is worth examining the interaction of immune cells with viruses of the herpes family, one of which is EBV infection. The highest incidence of this disease is among people between the ages of 5 and 25 years. The disease is usually asymptomatic and self-limiting, especially during early childhood^11^. Infectious mononucleosis syndrome is characterized by fever, pharyngitis and lymphadenopathy, often with elevated liver enzymes associated with hepatosplenomegaly^4,12,13^. The peripheral blood count shows leukocytosis (10 × 10^3^–20 × 10^3^/μl) with a significant number (more than 10%) of atypical monocytic lymphocytes, which correspond to the activated T cells^14^. The etiological factor in 90% of cases of typical mononucleosis is EBV, belonging to the Herpesviridae family^14^. Once infected, a person has B lymphocytes that contain the DNA of the virus throughout his/her life, in a latent phase^11^. Under certain conditions, EBV can be reactivated.

In response to the appearance of the pathogen, the body's defense system, the immune system, is activated^15^. Lymphocytes are the main cells involved in an acquired immune response. They have immunological specificity, recognize antigens and can react to them, transforming into cells capable of performing specific effector functions. B lymphocytes and their derived plasma cells produce immunoglobulins, which are Y-shaped proteins. Immunoglobulins are composed of two basic units: polypeptide heavy chains and light chains, covalently linked by disulfide bonds^16^. The antibodies bind specifically to a certain antigen found on the surface of altered cells. Specific EBV antibodies, produced by B lymphocytes during the primary humoral immune response are IgM^17,18^. Early IgM antibodies are secreted even before the development of B lymphocytes and are therefore of low affinity^19^. While early B-cell development is characterized by an orderly arrangement of the Ig heavy and light chain unit, Ig proteins play an important role in the regulation of B-cell development^20^. Immunoglobulin receptors (BCR) are produced on the surface of naive B lymphocytes in the initial phase of an immune response. BCR is the most characteristic surface marker of B-cells. B-cell receptors and antibodies bind specifically to antigens, triggering a cascade of events in these cells leading to their activation. During the secondary response (which peaks around 1–3 days after exposure to the antigen), only IgG is synthesized. As a result, the concentration of IgG antibodies may increases significantly. However, disturbances in the production of antibodies as well as the function of humoral response effectors, may be a factor in the pathogenesis of various diseases. EBV is associated with malignant transformation^11^.

In our research to date, we have gained some insight into the specificity of lymphocyte responses to viral infection^9,21^. A Two-Dimensional Correlation Spectroscopy (2D-COS) analysis revealed some early, initial details of lymphocyte activation^22^. Two-dimensional synchronous maps indicated the formation of a receptor-ligand complex, while 2-D asynchronous maps pointed to B-cell development and immunoglobulin formation. Recently, it has been pointed out that additional information on cells and tissues can be provided by characteristics related to changes in the polarizability of light^23,24^.

The aim of this study is to examine activity in the lymphocytes of patients diagnosed with infection with the EB (Epstein–Barr) virus and to compare these results with data obtained for a control group of healthy volunteers. In this study, a thorough analysis of B lymphocytes was performed, indicating changes during lymphocyte activation, which are spectroscopic markers, important in the course of the disease. Further information on the functioning of B cells is provided by the analysis of changes in light polarizability observed as a function of the duration of the disease. Therefore, changes in the degree of depolarization ratio of the immunoglobulin marker observed in the course of infectious mononucleosis are also studied, indicating additional specificity of EBV infection.

## Results

### Results of standard diagnostic tests

**Figure 1:**
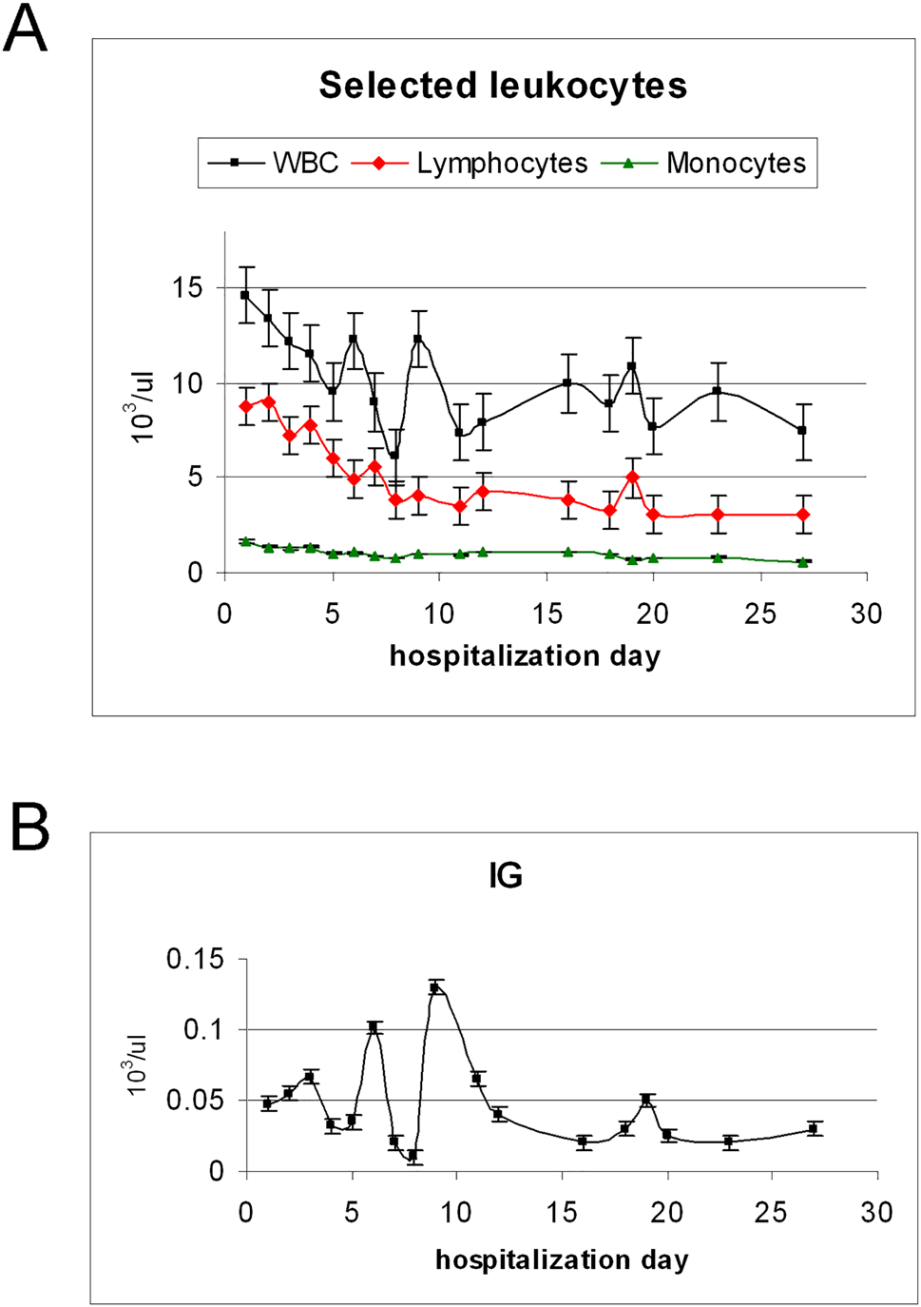
(**A**) Leukocyte count as the mean value for all patients in the study group: WBC [norm 4.00–10.00 × 10^3^/μl] (mean error bar equal to 3.14), lymphocytes [norm 0.80–4.00 × 10^3^/μl] (mean error bar equal to 1.66), monocytes [norm 0.16–0.80 × 10^3^/μl] (mean error bar equal to 0.30), (**B**) IG (immature granulocytes) level [norm 0.00–0.09 × 10^3^/μl] (mean error bar equal to 0.05), in patients diagnosed with infectious mononucleosis during consecutive days of hospitalization.

The patients whose lymphocytes were analyzed in this study were aged between 19 and 27 years. EBV-specific antibody tests were positive in all patients participating in the study (EBV anti-VCA IgM: positive). The blood count results are shown in Fig. 1. From the complete blood count, it could be concluded that the number of lymphocytes checked in patients with EBV was the highest in the first days of hospitalization, but also exceeded the values long considered normal at a later stage (see Fig. 1A). Another maximum is observed on day 7 of treatment and a less intense maximum is reached on day 19 of hospitalization. The number of lymphocytes tend to shift slightly in relation to the spectroscopic image.

Note the increased number of immature granulocytes after around a week of infection (see Fig. 1B). This increase in IG means that the immune response will be disrupted. EBV-induced hematopoietic changes suggest that viral-mediated immune suppression may occur, further exacerbating the pathology of EBV-associated disorders^25^.

The ALT level observed was depended on the condition of the individual patient, although these were usually well above normal.

### Raman microspectroscopic data

**Figure 2:**
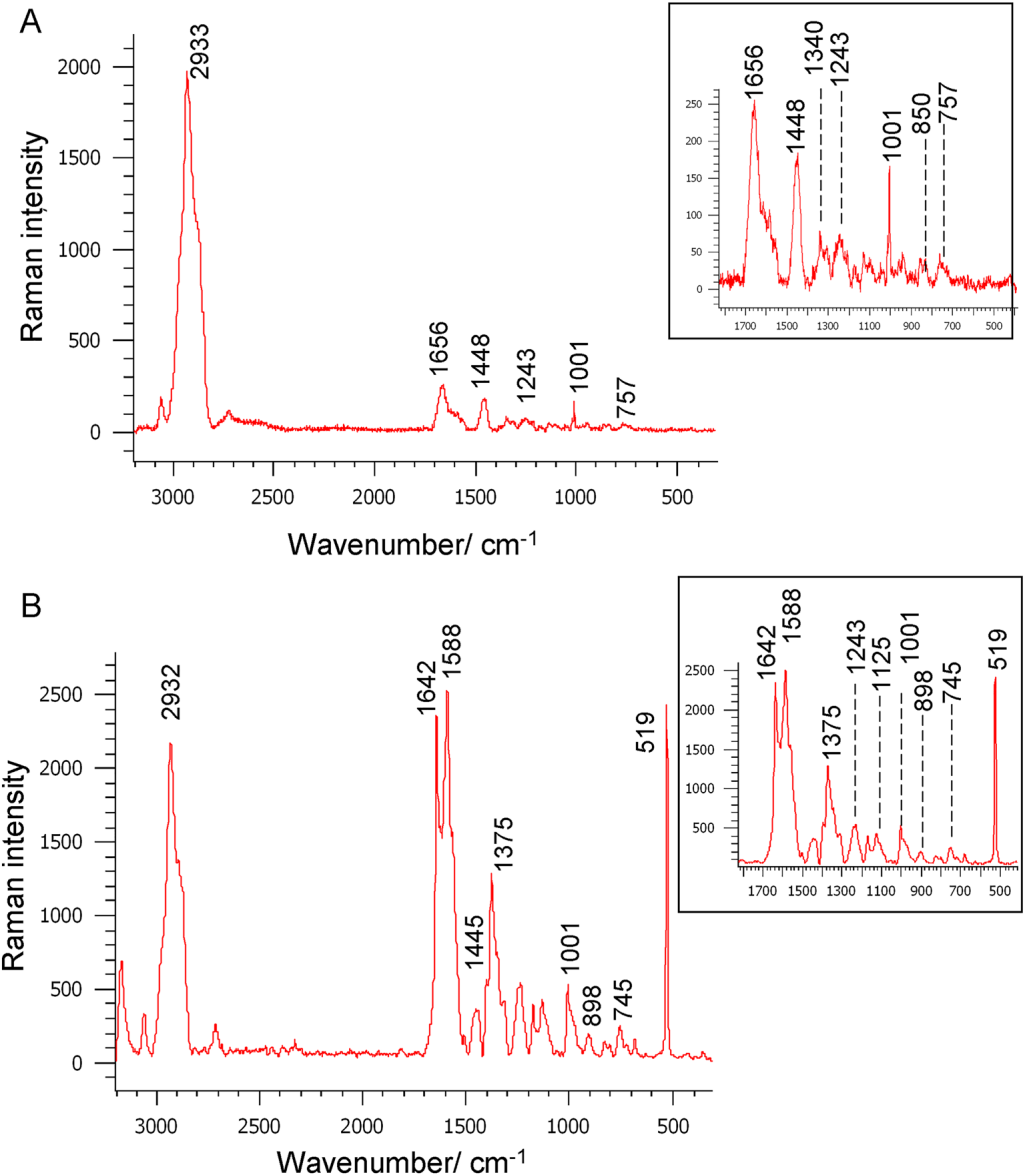
Average Raman spectrum of: (**A**) the naive lymphocytes from the control group, (**B**) the activated B-cell region of a representative patient on the first day of hospitalization, due to infectious mononucleosis, spectral range 3200–300 cm^−1^, laser line 514.5 nm. Insets show the magnified spectral region of 1700–500 cm^−1^. Statistical analysis was performed with PCA with Calibration 95.12744, Validation 87.87009 (Unscrambler X software packages, v. 10.3, CAMO Software, Oslo, Norway).

Figure 2 shows an example of the average Raman spectra of the naive lymphocytes of healthy volunteers. and from the activated region of the B-cell, the average Raman spectra of a representative patient diagnosed with infection with the EBV on the first day of hospitalization.

The spectral data clearly illustrate the biological material and changes related to its function. Noteworthy is the immunoglobulin marker band at position 520 cm^−1^, which undergoes spectacular changes during cell activation. EBV-transformed B-cells secrete immunoglobulins^14,20^. For comparison, lymphocyte measurements, taken from a control group of healthy volunteers who had never suffered from infectious mononucleosis previously indicate the absence or a very weak feature of the 520 cm^−1^ band (Fig. 2A). Other prominent Raman bands are observed in the spectra of B lymphocytes. which differ in terms of the location and/or intensity of the activated lymphocytes in the infectious mononucleosis and in healthy volunteers, and are specified in Table 1^6,7,9,21,22,26,27^.

**Table 1:** The main Raman bands (cm^−1^) and their assignments for the B lymhocytes of patients diagnosed with infectious mononucleosis and healthy volunteers (514.5 nm laser line)^6,7,9,21,22,26,27^.

Raman bands (cm^−1^)		Assignments
Infected	Healthy	
2932	2933	CH sym str of CH_3_ methyl groups, PC
2876	2881	CH asym str of CH_2_ methylene group
1665	–	Amide I (β conf)
	1656	Amide I (α conf)
1642		T
1588s	1584	G, A, nucleic acids; δNH_2_, Gln, Trp, Phe
1567	–	Glu
–	1448	G, A, nucleic acids, Phe, CH_2_ def., lipids
1375		A, CH_2_ def., lipids, PC
–	1336	Trp, CH def., endoplasmatic reticulum
–	1308	A,CH_2_ def., lipids,
1238	1243	Tyr, Amide III
	1173	Tyr, Ser
1169	–	G
1125	–	Asp, Glu, CC str., PE
1000	1001	Phe
898		A
822	850, 830	Tyr
–	757	Trp
745	–	T-ring breathing, nucleic acids, Trp
675	–	T, G, CS str
520	–	Disulfide bond

Band assignments may differ slightly if a different laser line is used.

*A* adenine, *G* guanine, *T* thymine, *C* cytosine, *Gln* glutamine, *Glu* glutamic acid, *Trp* tryptophan, *Tyr* tyrosine, *Phe* phenylalanine, *PC* phosphalidylcholine, *PE* phosphatidylethanolamine.

The appearance of the 520 cm^−1^ band, representing SS vibrations, indicates the activation of B lymphocytes, as a result of the secretion of immunoglobulins^17,21^. IgG antibodies contribute directly to the immune response, including the neutralization of viruses^16^. The immunoglobulin chains form the domains as complex three-dimensional structures. The structure of the immunoglobulins facilitates their bifunctionality: the variable region (V region) contains antigen-binding sites, and the fixed portion (C region) is the part of the molecule that is connected to cells. Raman spectroscopy is a well-established method of analyzing disulfide bonds, which are important bonds affecting the conformation and stability of proteins^28^.

**Figure 3:**
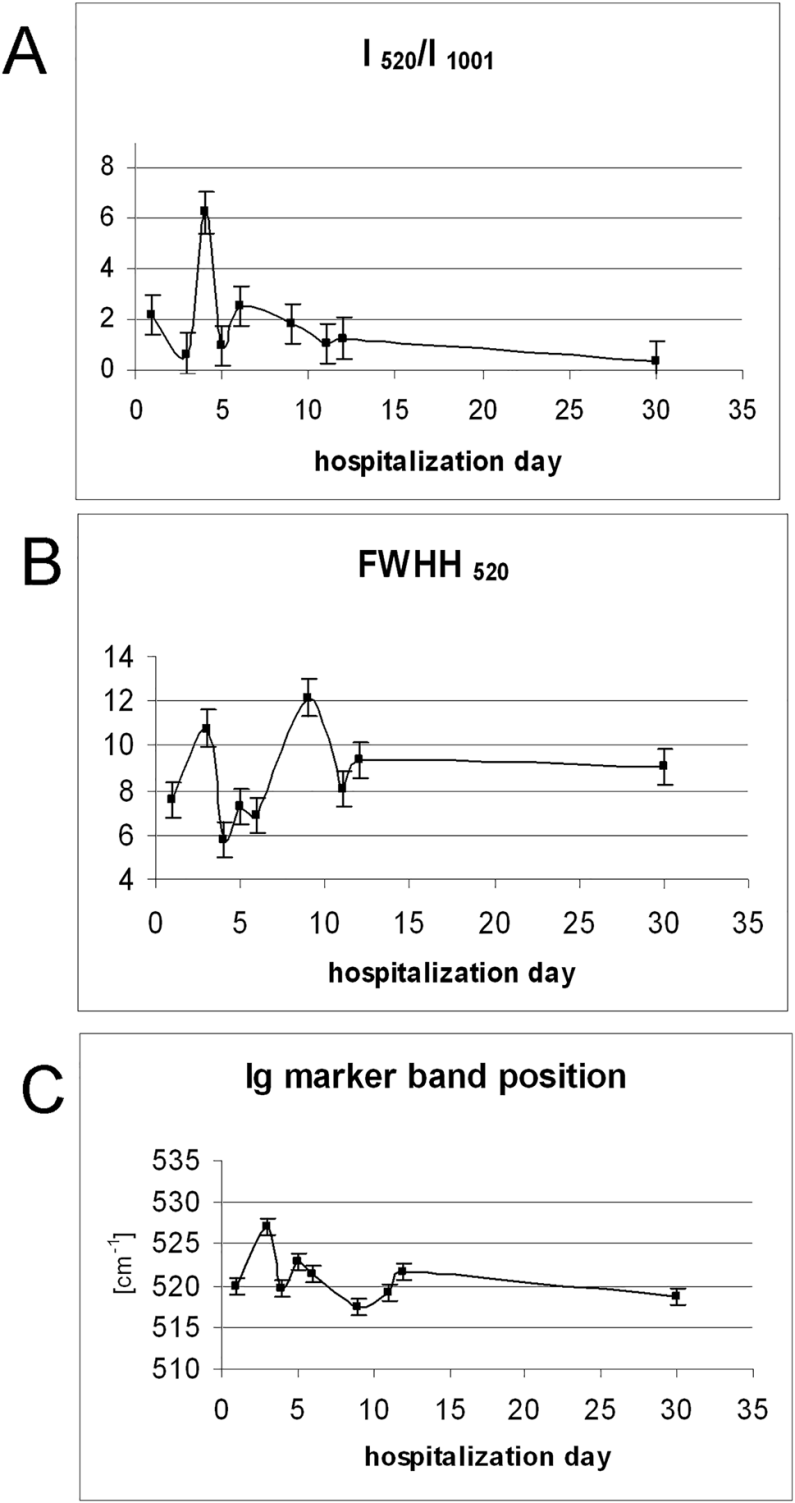
(**A**) Relative intensity (I_520_/I_1001_) (mean error bar equal to 1.51), (**B**) FWHH_520_ (mean error bar equal to 1.90) and (**C**) position of immunoglobulins marker band during hospitalization estimated as the mean for all patients (mean error bar equal to 0.55). The 520 cm^−1^ band parameters, i.e., intensity, position and half-width were evaluated using a Curve fit procedure in the WiRE factory software.

The SS band, observed at a wavenumber of approximately 520 cm^−1^ in the Raman spectra of patients diagnosed with infectious mononucleosis and consistent with Normal Mode Analysis, indicates the presence of the immunoglobulin gauche–gauche–trans (g–g–t) rotamer in the variable (VL) domain^29,30^. This assignment is also consistent with the presence of the CS band at 675 cm^−1^. Of note is the ratio of the intensity of the 520 cm^−1^ band to the 1001 cm^−1^ band, which was highest on the 4th day of hospitalization (equal to almost 6.5), (see Fig. 3A). This relative dependence of the intensity of the Ig marker band on the Phe band was obtained as the average of the spectra of all blood cells of each of the patients, measured on consecutive days of hospitalization. The highest intensity of the marker band was observed to correlate in time with its smallest half-width (see Fig. 3B). This indicates the appearance on the 4th day of hospitalization of one dominant conformation, characterizing the binding of SS immunoglobulins, which is adopted by activated lymphocytes and is enhanced as a result of increased secretion of IgG by activated lymphocytes^21^. Such a clear effect does not persist in the following days. The average ratio of the intensity of the 520 cm^−1^ band to the intensity of the 1001 cm^−1^ band, calculated for all activated cells on consecutive days, i.e., days 6, 12 and 30 of the disease, decreased to 2.5, 1.2 and 0.4, respectively (see Fig. 3A). This behavior indicates a weakening of the development of immunoglobulin secretion and may also indicate certain B-cell proliferative disorders caused by EBV-virus infection^31^.

The dynamics of changes in the full width at half-height (FWHH) of the characteristic Ig marker band, appearing in activated lymphocytes, is shown in Fig. 3B. FWHH has an increased half width on the 3rd and 9th days. This characteristic can be correlated with the change in the location of the Ig marker band, which is in the range of around 10 cm^−1^ (from 517 to 527 cm^−1^) and may be associated with the dynamically progressing, so-called affinity maturation, see Fig. 3C ^32^.

**Figure 4:**
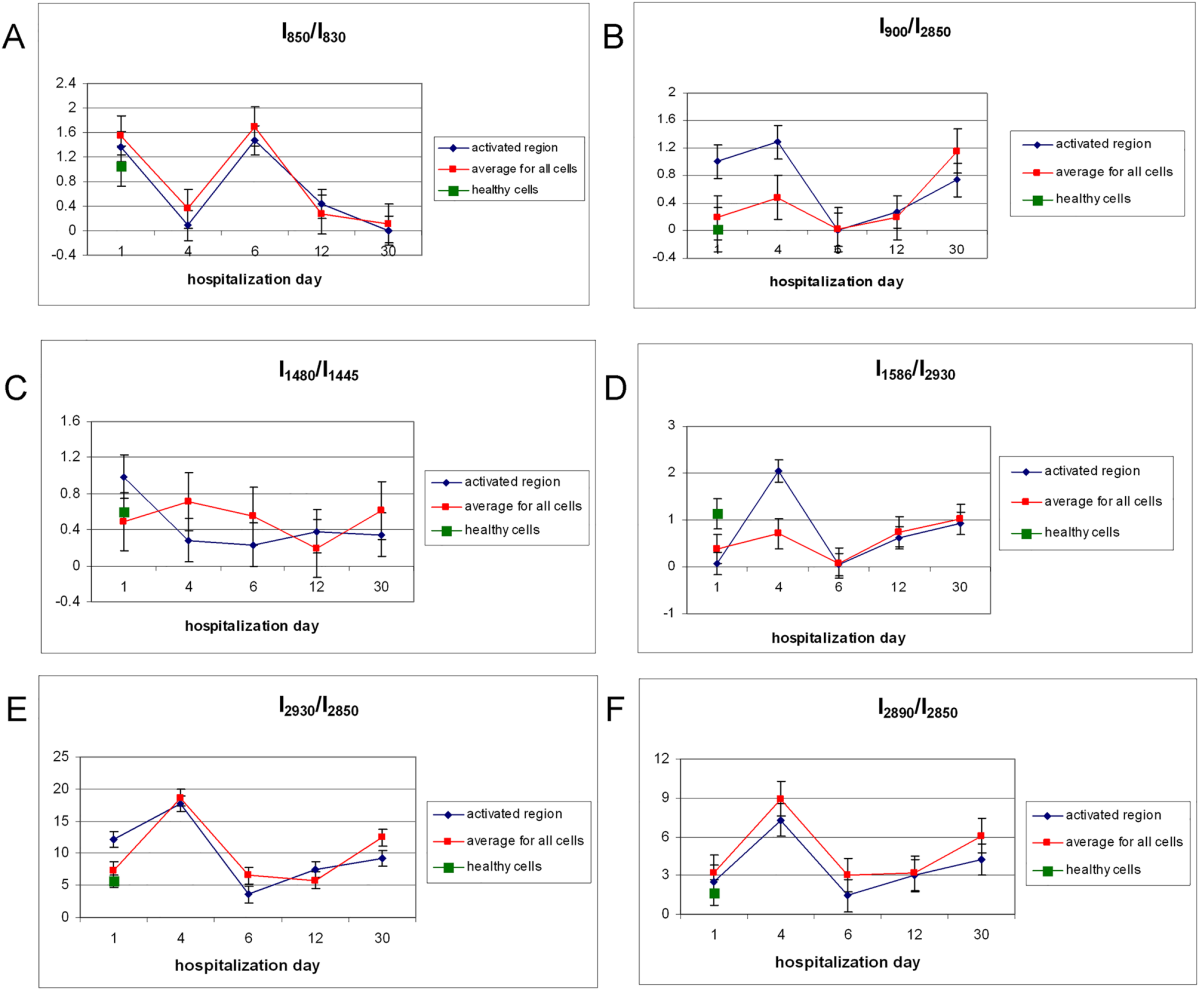
The (**A**) I_850_/I_830_ (SD equal to 0.30, 0.12, 0.20, 0.05 and 0.02 on subsequent measurement days, mean error bar equal to 0.14)._,_ (**B**) I_900_/I_2850_ (SD equal to 0.02, 0.02, 0.01, 0.01 and 0.02 on subsequent measurement days, mean error bar equal to 0.02)_,_ (**C**) I_1480_/I_1445_ (SD equal to 0.10, 0.10, 0.03, 0.03 and 0.02 on subsequent measurement days, mean error bar equal to 0.14)_,_ (**D**) I_1586_/I_2930_ (SD equal to 0.30, 0.20, 0.10, 0.20 and 0.10, mean error bar equal to 0.20)_,_ (**E**) I_2930_/I_2850_ (SD equal to 0.22, 0.19, 0.11, 0.10 and 0.10 on subsequent measurement days, mean error bar equal to 0.14) and (**F**) I_2890_/I_2850_ (SD equal to 0.14, 0.10, 0.10, 0.05 and 0.10 on subsequent measurement days, mean error bar equal to 0.10) intensity ratios for a representative patient with infectious mononucleosis, calculated from the activated B-cell region (red line) and obtained as the average of all B-cells spectra of all patients, measured during their hospitalization (dark line) on the 1st, 4th, 7th, 12th, and 30th day of hospitalization. Appropriate average values of intensity ratios for healthy volunteers are marked in green.

Figure 4 shows the analysis of the B-cells Raman spectra by presenting the intensity ratios of the selected bands in the course of hospitalization. The intensity ratios of the individual bands, presented as a function of time, were estimated from the averaged spectrum of the activated lymphocyte areas of a representative patient, as well as from the averaged B lymphocyte spectrum for all patients.

B-cell behavior is stimulated and controlled to a large extent by the B-cell antigen receptors, however, BCR signaling requires multiple steps, including tyrosine kinases as cellular regulating elements^20,32,33^. Tyrosine phosphorylation is one of the earliest events following BCR activation^34^. In the intact lymphocytes, a Tyr doublet of bands, with comparable intensities, appeared at a position of around 850 cm^−1^ and 830 cm^−1^ (see Fig. 2A). These bands are a result of Fermi resonance between the breathing vibration of the benzene ring and the overtone of the bending vibration outside the plane of the atoms of this ring in the *para* position^35^. In activated lymphocytes, the intensity of these bands changes (see Fig. 4A). On the 4th day of hospitalization, the intensity of the band around 830 cm^−1^ increases. This feature indicates that Tyr residue (associated with the cell surface BCR complex) is involved in the Tyr phosphorylation process^36^. Noncovalently associated components of the cell surface BCR complex and Igα and Igβ cytoplasmic tails carry immunoreceptor tyrosine activation motifs (ITAMs)^18^. Thus, the increase in the average intensity ratio of the 830 cm^−1^ band to the intensity of the 850 cm^−1^ band on the 4th day of hospitalization means a decrease in the intensity of the I_850_/I_830_ ratio shown in Fig. 4A and signifies an initiation of BCR signaling and B-cell activation^20^. This process is also indicated by an increase in the tyrosine marker band, 1586 cm^−1^ demonstrating its phosphorylation, see Fig. 4D ^36^.

Upon contact with the virus, the intensity of the individual bands changes markedly, representing the stimulated proliferation of lymphocytes into a clone of identical cells. Thus, the band of 1370 cm^−1^ in activated B-cells indicates CH_2_ wagging of PC and thus the formation of membrane phospholipids^26^. At the same time, the 745 cm^−1^ nucleic acid marker band increases. The 1665 cm^−1^ band of amide I (conf. -β), characterizing the secondary structure of Ig also increases, while the band of amide I (conf.-α; 1656 cm^−1^) decreases (see Table 1).

An analysis of CH stretching vibrations allows an estimation of the CH_2_ groups’ alteration and changes in the lipid system, caused by the introduction of viral proteins into the cell^37^. B-cells show distinct bands at approximately 2850 cm^−1^, 2890 cm^−1^ and 2930 cm^−1^ and are fainter at approximately 2960 cm^−1^, corresponding to the symmetric and asymmetric stretching vibrations of the CH_2_ and CH_3_ group, respectively^26^. The band around 2890 cm^−1^ is sensitive to the environment, reflecting its influence on the ordering of methylene groups. The observed intensity ratio of 2890–2850 cm^−1^ reveals a measure of lateral packing, allowing changes in the ordering of methylene groups and chains to be traced, since the 2850 cm^−1^ band is relatively stable. The highest value occurs on day 4 and is around 9, which indicates the greatest changes taking place in the cell, see Fig. 4F. Following a decrease, observed after around a week, this rate increases again by the 30th day of hospitalization, indicating further changes in the cell.

Another marker band, the 2930 cm^−1^ band of the CH stretching vibration of the CH_3_ groups, varies significantly with regard to changes in the proteins in the terminal groups; its intensity ratio was calculated relative to 2850 cm^−1^. The trend of changes is similar to the previous case. It reached the highest value on the 4th day of hospitalization, then lowered and finally increased on the 30th day of hospitalization (see Fig. 4E).

An increase in the intensity of the signal associated with the proliferation of proteins and a simultaneous, less intense signal from immunoglobulins may indicate the stimulus of the latent membrane protein, 2A and 2B (LMP) of the viral protein EBV in B-cell proliferative disorder^34^. The specific features of LMP2A include an alteration of the typical BCR signal transduction in B-cells by downregulation of tyrosine phosphorylation. LMP2A affects apoptosis by disrupting and dysregulating apoptotic signals^38^. The characteristic LMP2A terminal domain motif, containing the Pro-Tyr amino acid residues (monitored by the Raman ratio of the Pro I_900_/I_2850_ marker bands), was significant on the 4th day of hospitalization and then markedly increased on day 30, whereas it was equal to zero in the case of the healthy volunteers (see Figs. 2B, 4B)^39,40^. These interactions were also confirmed by the scissor vibrations of the CH_2_ group in Pro, observed at a position of around 1450–1460 cm^−1^ (see Fig. 2). The intensity of the 1445 and 1480 cm^−1^ bands (estimated by the I_1480_/I_1445_ intensity ratios) is distinctly different for the average of all cells and for the average of the activated cell area, see Fig. 4C. An increase in the average intensity of the band around 1480 cm^−1^ in the area of the whole cell during the first week of hospitalization indicates the involvement of the C=O imide site of proline in a fairly strong hydrogen bond in the protein^41^. The variability of the I_1480_/I_1445_ ratio throughout the course of the disease indicates changes in the binding conformation in which Pro is involved.

### Analysis of the size of the activated B-cell surface

**Figure 5:**
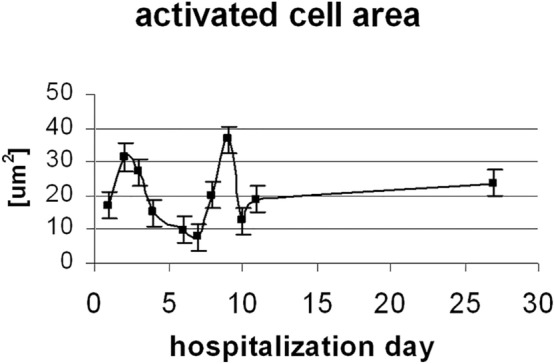
Activated B-cell region (μm^2^), assessed by the appearance of the 520 cm^−1^ marker band in a given cell fragment, as a function of hospitalization time (mean error bar equal to 3.97).

During activation, the cell shape and morphology change significantly. On the first day of the disease, cells are extensively enlarged and have irregular shapes^42^. The dependence of the size of the B lymphocyte on the proliferative response was also noticed by other authors^42–44^. However, B-cells can differentiate in a variety of ways^32^. The assessment of B-cell activation was also performed by evaluating the activated areas in the cells, defined by the presence of the Ig marker (shown in Fig. 5). A marked increase in the activated area is observed at two time intervals, around the 3rd and the 9th day, which seems to indicate the development of activated plasma B-cells, an increased production of antibodies to fight the virus^9,21^. An increase in the activated cell area around the 9th day of hospitalization seems to be consistent with B-cell transformation and differentiation into antibody-producing plasma cells, the highest concentration of which is expected around the 2nd to the 4th week of illness^12^. However, B-cell transformations can also occur in other ways, such as differentiation into germinal center (GC) B-cells, or memory B-cells^34^. A clear increase in the half-width was also found around the 3rd and 9th day of hospitalization (see Fig. 3). This may indicate the process of transformation of B lymphocytes and the creation of an EBV reservoir in them^14^.

### Polarization measurements

**Figure 6:**
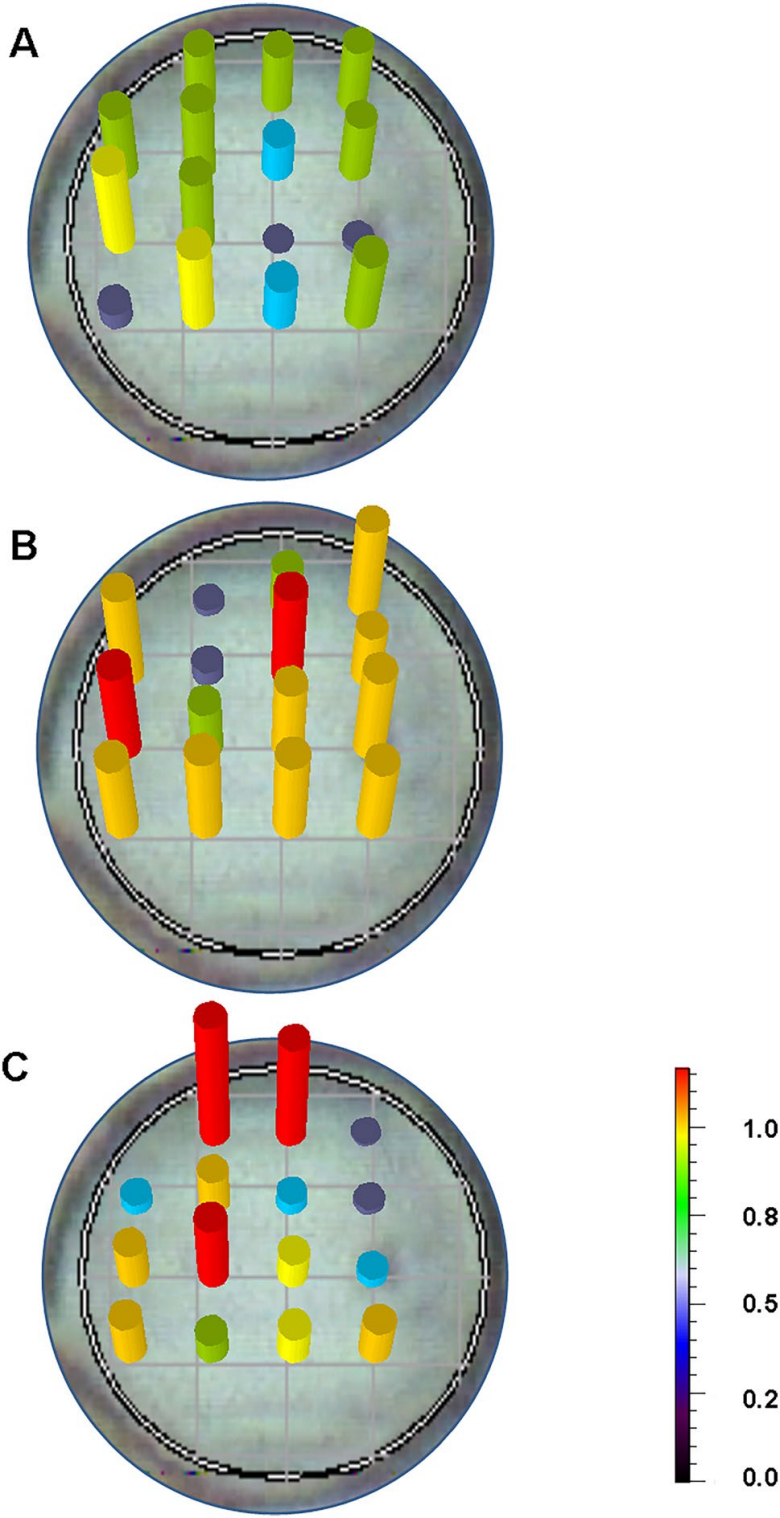
Depolarization ratio (ρ = I_⊥_/I_||_, where I_||_ and I_⊥_ are the intensity of scattered light, parallel and perpendicular to the polarization of the incident laser light, respectively) obtained for the B cells of a patient diagnosed with infectious mononucleosis on (**A**) 4th, (**B**) 6th, and (**C**) 9th day of hospitalization.

Additional information on the tested system was provided by the Raman measurement of the depolarization ratio^45^. The laser beam incident on the sample was polarized. Measurements of the degree of depolarization were taken, and scattered radiation at two positions of the polarizer in relation to the incident light was analyzed. The polarization of scattered radiation was measured in a perpendicular and parallel direction to the polarization of the excitation laser light. The depolarization ratio of the 520 cm^−1^ band, an immunoglobulin marker, was analyzed in the course of infectious mononucleosis (ρ = I_⊥_/I_||_, where I_||_ and I_⊥_ are the intensity of scattered light parallel and perpendicular to the polarization of the incident laser light, respectively) (see Fig. 6).

Polarization measurements, at the onset of infection characterize the initial response to the presence of the virus, represents the initial development of B-cells characterized by a uniform immunoglobulin arrangement (see Fig. 6A). In the following days of hospitalization, the spectra obtained show an inverted polarization for the 520 cm^−1^ band in many areas in the activated B cell. This puzzling characteristic appears especially at a time when the patient should be recovering (see Fig. 6B, C). This may indicate the presence of a different immunoglobulin conformation, e.g., some B-cell proliferative disorder, possibly indicative of a transition to the latent form^31^. In the later stage of the disease, the influence of the latent membrane protein LMP2A is more pronounced, which lowers the threshold required for mediated B-cell proliferation. The spectroscopic picture that reflects this disruption of signaling at molecular level reflects the anomalous degree of depolarization observed in the B lymphocytes studied, but also in some other biological systems^46^.

## Discussion

Raman microspectroscopy is a modern method for detecting changes in individual blood cells^1,2,6^. Therefore Raman spectroscopy is chosen to try to get an alternative insight into the mechanism of infectious mononucleosis.

The experimental material included blood samples which is obtained from patients diagnosed with infectious mononucleosis and, for comparison, from healthy volunteers. It is found that the 520 cm^−1^ band indicates the formation of immunoglobulins, so it could be a biomarker of lymphocyte cell activation^9,21^. During cell contact with the antigen increased protein production and increased expression of immunoglobulins are monitored (see Fig. 2B).

The highest intensity of the marker of immunoglobulin activation is observed on the 4th day of hospitalization, while the greatest increase in the number of lymphocytes is noticed at the beginning of hospitalization (see Fig. 3A and Fig. 1A). No activated lymphocytes are observed in the blood of healthy volunteers (see Fig. 2A). It is emphasized that B lymphocytes play a special role in viral infection, producing antibodies that can neutralize and remove the virus before it enters the cell^47^. B lymphocytes are the main target of the EBV, which is strongly associated with certain B-cell malignancies^48^. The symptoms of latent infection and the influence of LMP2A on B-cell signal transduction in the course of infectious mononucleosis is noted and evaluated^49^. And signal transduction in the cell is important because it determines the level of Ig production, and consequently, virus replication inhibition^47^.

Polarization measurements indicate structural changes in protein domains. The anomalous degree of depolarization, obtained over the course of the disease, may be due to the influence of the viral protein, LMP2A, on the dysregulation of receptor signal transduction. Testing the degree of depolarization of the immunoglobulin marker, observed in the course of infectious mononucleosis, may indicate an additional specificity of EBV infection and provide supplementary information on the infection and its course. The obtained results indicate that the search for a new conformation of Ig, which is expressed as a broadening of the Ig marker band and a shift in the marker band positon, indicates dysregulation of signal transduction by the virus (see Fig. 3B, C). These relationships is considered as a characteristic activity of the EBV virus in relation eg. to the influenza virus^21,32,47^. These spectroscopic facts correlate with lymphocyte proliferation and with the increase in the number of immature granulocytes observed in medical tests (see Fig. 1B).

Early B-cell development is characterized by an orderly rearrangement of Igα and Igβ heterodimers that combine with surface immunoglobulin to form BCRs^50,51 ^. These orderly changes are confirmed by the spectroscopic results, by the distinct formation of the Ig marker position, its intensity and the small half-width observed on the 4th day of hospitalization (see Fig. 3).

The cross-linking of surface immunoglobulins leads to the activation of kinases. Tyr phosphorylation occurs in most processes monitored using spectroscopic indicators of marker band intensity ratios, it is observed in the activated area of the B-cell, but also in the case of average values for the whole cell (see Fig. 4).

Around a week after diagnosis, new spectral features appear. These include a clear decrease in the intensity of the Ig marker and shifts in its position, which indicates a change in the Ig conformation pattern. This pattern matching is indicated by polarization measurements and the anomalous degree of depolarization observed in the increasing area activated in B-cells during the course of the disease (see Fig. 6). The study results can help predict the severity of the infection, which has important clinical implications.

## Methods

### Blood samples

The blood of 20 patients, diagnosed with infectious mononucleosis (treated in the Department of Infectious Diseases at the University Hospital in Kraków) was analyzed. Patients’ blood was collected during hospitalization for a control diagnostic test, up to the 30th day after diagnosis. The measurement stages were the corresponding days of blood collected: 1st, 4th, 6th 12th and 30th day of hospitalization, then analyzes were carried out using the Raman spectroscopy method. In addition, blood from healthy volunteers who had never suffered from infectious mononucleosis was analyzed for comparison.

The MACSxpress Whole Blood B-cell isolation technology, provided by Miltenyi Biotec GmbH, was employed for the separation of the lymphocytes’ subset directly from anticoagulated human whole blood. An isolation mix was made from kit components and was freshly prepared before the cell separation procedure. The magnetically labeled cells adhered to the wall of the tube and were carefully collected while the tube was still in the magnetic field..

The studies were carried out in accordance with the guidelines for good clinical practice (GCP), in accordance with the principles of ethics of medical research, involving people as defined by the Helsinki Declaration. All studies were approved by the Bioethics Committee of the Jagiellonian University (Opinion No 122.6120.112.2015 from 25.06.2015). Informed consent was obtained from all subjects involved in the study.

### Raman micro-spectroscopic method

Raman spectroscopy measurements were made using a mapping technique with a Renishaw InVia spectrometer coupled with a Leica optical microscope (using a 100 × objective lens) in the spectral range of 3200–300 cm^−1^. The samples were excited with an argon laser beam, with a wavelength of 514.5 nm, the laser power to which the that samples were subjected was around 1–3 mW.

The assignments of the relevant Raman bands are summarized in Table 1.

### The standard diagnostic tests

The ELISA test was used as the basic diagnostic test to confirm the presence of the infection^17,52,53^. EBV-specific antibody testing is performed to confirm acute EBV infection^51^. The acute phase of infectious mononucleosis is characterized by rapid IgM and IgG antibody responses to the viral capsid antigen (VCA) in all cases and an IgG response to the early antigen (EA) in most cases. In addition, the level of alanine aminotransferase (ALT) is usually significantly higher among patients with infectious mononucleosis, compared to the control group^54^.

## Data Availability

All the generated data and the analysis developed in this study are included within the article. Raw data cannot be shared due to patient confidentiality.
